# A Novel Case of *NOTCH1* Variant and Nonimmune Hydrops Fetalis: A Case Report

**DOI:** 10.1155/crig/6865742

**Published:** 2025-09-01

**Authors:** Genevieve R. Mazza, Alesandra R. Rau, Madushka Y. De Zoysa

**Affiliations:** ^1^Division of Maternal-Fetal Medicine, Department of Obstetrics & Gynecology, University of California, Irvine, California, USA; ^2^Fetal Care Center of Southern California, Children's Hospital of Orange County (CHOC), Orange, California, USA

**Keywords:** case report, fetal hydrops, nonimmune hydrops, *NOTCH1* pathogenic variant, perinatal outcome

## Abstract

Nonimmune hydrops fetalis (NIHF) refers to the pathologic accumulation of fluid within the fetus due to causes other than red cell alloimmunization and now accounts for up to 90% of fetal hydrops cases. Fetal hydrops is associated with significant morbidity and mortality, and the exact prognosis is largely dependent on the underlying etiology. The most common etiologies include cardiovascular causes and chromosomal or genetic abnormalities. Despite this, diagnostic testing with karyotype or chromosomal microarray only identifies approximately 25% of cases, and up to 20% of cases remain idiopathic or unknown. We report the first known case of NIHF related to a *NOTCH1* pathogenic variant. In this case, NIHF was diagnosed at 30 weeks' gestation in a fetus with low-risk prenatal genetic screening, noncontributory anatomic survey, and normal chromosomal microarray. The hydrops was uniquely localized to scalp edema and pleural effusions requiring bilateral thoracentesis and never progressed to involve pericardial effusion or ascites. Whole exome sequencing diagnosed a novel pathogenic variant in the *NOTCH1* gene. This is the first reported case of NIHF in the setting of *NOTCH1* pathogenic variant and is an important addition to the existing literature on this incredibly diverse, high-risk pathology.

## 1. Introduction

Hydrops fetalis refers to the pathologic accumulation of fluid within the fetus and is associated with significant neonatal morbidity and mortality. It is defined by at least two abnormal fluid collections in the fetus that include ascites, pleural effusion, pericardial effusion, and skin edema. It is also associated with polyhydramnios and placentomegaly [1, 2]. Nonimmune hydrops fetalis (NIHF) specifically refers to cases not attributed to red cell alloimmunization and accounts for up to 90% of cases [2]. The prognosis is largely dependent on the underlying etiology, the gestational age at diagnosis, and the gestational age at delivery [2]. Thus, a search for the cause of NIHF is imperative to guide patient counseling, treatment planning, and delivery preparations [3].

While the etiologies of NIHF are diverse, the clinical presentation is an imbalance in the movement of fluid between the vascular and interstitial spaces, due to either an increase in hydrostatic capillary pressure, reduction in plasma oncotic pressure, obstruction of lymphatic flow, or damage to capillary integrity [1, 4]. The underlying cause can be cardiovascular, chromosomal or genetic, structural, hematologic, urologic, or neurologic, among many others [1, 2]. Diagnostic testing with karyotype or chromosomal microarray only identifies about 25% of cases, and despite a thorough search for an underlying cause, approximately 20% of cases remain idiopathic or unknown [5, 6].

We present a novel case of NIHF in a patient whose prenatal workup, including a detailed anatomic survey, fetal echocardiogram, and invasive prenatal testing, failed to identify an underlying cause. The purpose of this case report is to highlight the growing etiologies of NIHF and the clinical utility of Whole exome sequencing (WES). All the patients allowed personal data processing, and informed consent was obtained from all individual participants included in the study. As the patient is a minor, written informed consent was provided by the parent.

## 2. Case Report/Case Presentation

**Figure 1:**
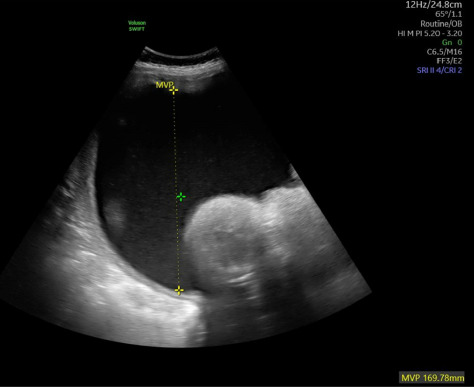
Ultrasound demonstrating severe polyhydramnios, with a maximum vertical pocket of 16.9 cm.

**Figure 2:**
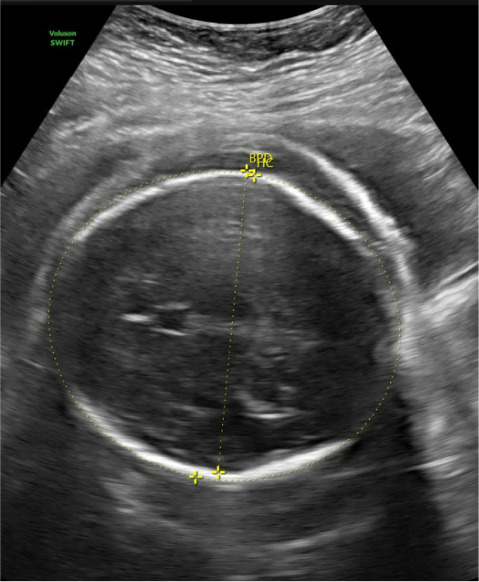
Ultrasound demonstrating scalp edema.

**Figure 3:**
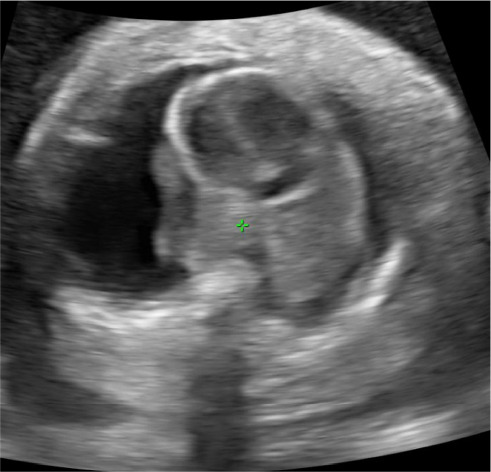
Ultrasound demonstrating bilateral pleural effusions.

A 37-year-old Gravida 2 Para 1 was well known to the obstetrics service after establishing prenatal care at 8 weeks' gestation. Her pregnancy was complicated by advanced maternal age, Class 1 obesity, and prediabetes. She had low-risk noninvasive prenatal testing (NIPT), normal maternal serum alpha-fetoprotein (msAFP), and normal nuchal translucency measurement at 1.9 mm. However, at the time of her nuchal translucency ultrasound, there was a concern for abnormal heart views. She was seen by pediatric cardiology and completed a fetal echocardiogram, which demonstrated a right-sided aortic arch, right ductal arch, and a small ventricular septal defect. At 31 weeks' gestation, a growth ultrasound noted new findings of fetal hydrops due to scalp edema and large bilateral pleural effusions with concurrent polyhydramnios (amniotic fluid index of 34.1 cm). Notably, there was no ascites or pericardial effusion, and the skin edema was limited to the scalp (Figures 1, 2, and 3). This hydrops was not explained by the known cardiac anomalies. Thus, she was admitted to the antepartum unit at a large academic medical center, where she received betamethasone, genetics consultation, and expedited workup of the new hydrops. Maternal labs demonstrated Rh positive status with negative antibody screen, rubella immunity, and negative TORCH panel aside from incidental herpes simplex Type 1 (HSV-1) IgG positivity. Middle cerebral artery Dopplers were normal, thus fetal anemia was not suspected. An amniocentesis and bilateral fetal thoracenteses were performed without complication. The amniotic fluid was sent for chromosomal microarray, Noonan spectrum disorders/RASopathies panel, as well as infectious testing for cytomegalovirus (CMV), parvovirus B19, and *Toxoplasma gondii*. Cell count performed on the fetal pleural fluid demonstrated 97% lymphocytes. The patient was subsequently discharged home. Labs ultimately resulted in a normal female microarray, a negative Noonan/RASopathies panel, and negative PCR testing for CMV, parvovirus B19, and *Toxoplasma*.

**Figure 4:**
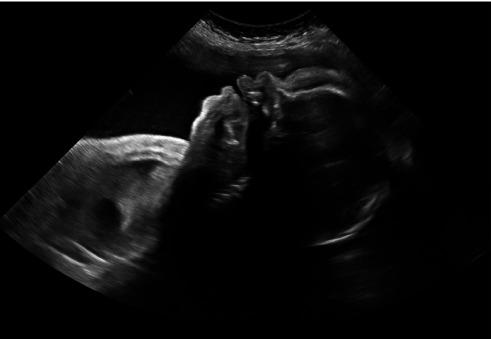
Ultrasound demonstrating worsening scalp and neck edema.

The patient returned to labor and delivery at 32 weeks' gestation with symptoms of worsening abdominal distention, shortness of breath, and orthopnea. On ultrasound, she was found to have reaccumulation of severe polyhydramnios and bilateral fetal pleural effusions as well as worsening scalp and neck edema (Figure 4) The patient was then readmitted for amnioreduction, closer monitoring of maternal and fetal status, and neonatal intensive care consultation. Amnioreduction was performed for the patient's severe symptomatic polyhydramnios without complication. WES was requested after a repeat genetics consultation, which ultimately resulted in heterozygosity for a pathogenic variant in the *NOTCH1* gene.

In collaboration with the neonatal intensive care team, the patient, and her partner, we reviewed the options for continued treatment with repeat fetal intervention versus planned preterm delivery. Ultimately, a plan was made for delivery at 34 weeks' gestation due to progressive fetal hydrops despite fetal interventions, risk for in-utero fetal demise, and concern for potential development of maternal complications. The mode of delivery was planned to be via primary cesarean section with concurrent bilateral fetal thoracentesis immediately prior to delivery to support the neonate's cardiorespiratory stabilization after birth. Prior to planned delivery, the patient received a rescue course of betamethasone at 33 weeks' gestation due to fetal heart rate decelerations.

At a gestational age of 34 weeks and 0 days, a viable female infant was delivered via primary cesarean section, with APGARs of 5 and 8, weighing 2770 g. As planned, bilateral thoracentesis was performed in the operating room just prior to cesarean delivery with 40 and 44 cc of fluid removed from the left and right pleural spaces, respectively.

**Figure 5:**
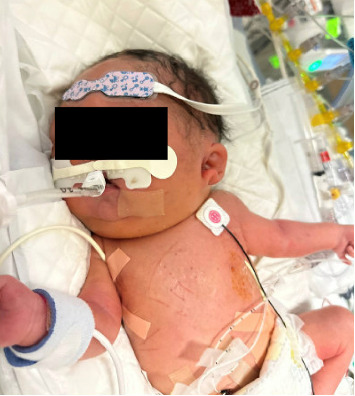
Immediately after delivery, demonstration of severe scalp and neck edema with sparing of the lower extremities.

After birth, the infant was intubated in the delivery room and admitted to the neonatal intensive care unit (NICU). Bilateral chest tubes were ultimately placed due to left-sided pleural effusion and right pneumothorax. Physical exam of the infant noted significant edema in the scalp, face, and neck, with moderate edema extending to the bilateral arms and hands (Figure 5). There was no significant edema affecting the lower extremities. Postnatal echocardiogram demonstrated a right aortic arch and left-to-right flow across a moderately patent ductus arteriosus, moderate right ventricular hypertension, and turbulent flow in the superior vena cava. Persistent chylous drainage from chest tubes and edematous head and upper extremities were concerning for obstruction of venous collaterals versus a primary lymphatic disorder. Ultimately, the neonate was transferred to our affiliated Children's Hospital, where a cardiac catheterization demonstrated a severe narrowing of the left innominate vein and occlusion of the right internal jugular vein. Balloon dilation was performed on the left innominate vein, and postprocedure, resolution of chylothorax was noted with removal of chest tubes and significant improvement in head and neck edema. Ultimately, the newborn was extubated and discharged home at 11 weeks of life (corrected age 5 weeks).

## 3. Discussion

To our knowledge, this is the first case of NIHF in the setting of *NOTCH1* pathogenic variant. Specifically, this was believed to be a de novo variant that results in a frameshift mutation in the *NOTCH1* gene and premature protein truncation. This variant has not been reported in the existing literature or in population databases. The *NOTCH1* gene is located on Chromosome 9 and encodes a protein that functions as a transmembrane receptor [7, 8]. When cleaved, this receptor migrates to the nucleus to modulate gene expression and plays an important role in determining cell fate during development [8]. It has been implicated in numerous types of congenital heart disease including bicuspid aortic valve, aortic stenosis, hypoplastic left heart syndrome, coarctation of the aorta, and tetralogy of Fallot, as well as Adams–Oliver syndrome and T cell acute lymphoblastic leukemia [7–9].

Although the exact mechanism behind the development of nonimmune hydrops in the current case is not fully elucidated, the proposed mechanism involves an abnormality in the lymphatic system compounded by abnormal venous return secondary to severe constriction of major head and neck vessels, which may also be a secondary phenotype of the mutation. Prior studies have demonstrated the important role of *NOTCH1* signaling and lymphangiogenesis [10–12]. Lymphatic endothelial cells (LECs) are affected by the NOTCH pathway [10]. Fatima et al. demonstrated that *NOTCH1* is an important regulator of LEC spouting and growth in the developing mouse model such that LEC-specific deletion of *NOTCH1* resulted in lymphatic overgrowth in mice. The authors ultimately concluded that NOTCH signaling is vital to proper lymphangiogenesis during embryonic development [11]. Zheng et al. support this finding by demonstrating a synergistic relationship between NOTCH suppression and VEGF in inducing lymphangiogenesis in vivo and in vitro. The authors assert that the NOTCH pathway is an important negative regulator of lymphatic sprouting, thus exerting important effects on lymphatic endothelial quiescence [12]. It is possible that in our case of NIHF, the *NOTCH1* pathogenic variant led to premature protein truncation, causing a nonfunctioning gene resulting in pathologic lymphangiogenesis.

Although in mouse models, there is a demonstrated relationship between *NOTCH1* mutations and defective lymphatic system development [10, 11], review of the current literature yields no documentation of an association between NOTCH1 pathogenic variants and fetal hydrops. In a large series of 127 cases of NIHF, the authors sought to establish the diagnostic yield of exome sequencing in the setting of unexplained hydrops. They included cases with a nondiagnostic karyotype or chromosomal microarray and ultimately identified diagnostic genetic variants in 29% of fetuses utilizing exome sequencing. These variants caused a wide array of disorders. Importantly, none of the identified variants involved *NOTCH1* [6]. An additional comprehensive review of chromosomal abnormalities associated with fetal pleural effusions did not identify *NOTCH1* pathogenic variant [13]. This reinforces the novelty of our case and the importance of increasing awareness for this genetic variant's association with nonimmune hydrops.

In conclusion, NIHF carries a guarded prognosis that is largely dependent on the underlying etiology. Conventional methods often fail to provide a diagnosis. WES provides an important diagnostic advantage, which can aid clinicians in patient counseling and appropriate antenatal and postnatal management. This case of a *NOTCH1* pathogenic variant is an important addition to the growing body of literature on nonimmune hydrops as well as the embryonic manifestations of this genetic defect.

## Data Availability

The data that support the findings of this study are available from the corresponding author upon reasonable request.
